# Disposable Paper Cups: A Study on Potentially Toxic Elements, Radiological Impact, and Feasibility of Valuable Elements Recovery

**DOI:** 10.3390/toxics13030179

**Published:** 2025-02-28

**Authors:** Mahmoud Mohery, Ahmed Mindil, Sheldon Landsberger, Mohamed Soliman

**Affiliations:** 1 Department of Physical Science, College of Science, University of Jeddah, Jeddah 80327, Saudi Arabia; amindil@uj.edu.sa (A.M.);; 2 Nuclear Engineering Teaching Lab Walker Department of Mechanical Engineering, University of Texas at Austin, Austin, TX 78712, USA; s.landsberger@mail.utexas.edu

**Keywords:** paper and plastic cups, single use products, radiological impact, potentially toxic elements, neutron activation, valuable elements recovery

## Abstract

This study characterizes single-use paper cups with respect to potentially toxic elements, radiological impact, and the potential of economic metals recovery from incineration residue. Thirty-six elements were identified in the analysis of paper cups, including naturally occurring radionuclides ^40^K, Th, and U, as well as potentially toxic elements such as Al, Ba, Co, Cr, Cu, Mn, Mo, and V using neutron activation analysis. The determined mass fractions varied significantly, with Mg, Al, and Ca present in notably high concentrations. A comparison with plastic cups revealed higher mass fractions of most elements in paper cups. The study also evaluated the potential for valuable element recovery from the incineration ash of paper cups. It demonstrated a promising potential for recovery of Cu—especially from blue and green cups—and Mg, as their mass fractions are above the ore cut-off grade. The amount of CO_2_ emissions from the incineration of paper cups was estimated at 1.77 kg/kg. The activity concentrations of ^40^K, Th, and U were estimated in the incineration ash, with ^40^K accounting for around half of the total detected activity. The median radioactivity was 35 Bq/kg. Although the radioactivity levels are low, they should be considered due to the large volume of paper cup waste. The radiological risk was assessed using various hazard indices, indicating minimal risk to human health. The dose rate and the annual dose are well below the recommended limits, and the excess lifetime cancer risk (*ELCR*) of 2.17 × 10^−5^ is well below the typical safety limit.

## 1. Introduction

Paper and paperboard products are the most preferred and common materials in food packing processes [1]. Their popularity stems from their desired characteristics, as they are affordable, easily available, lightweight materials, flexible, and serve as a good barrier to oxygen, microbial entities, and moisture. Disposable paper cups are the most commonly used as beverage containers. They are used globally in most restaurants, coffee and tea shops, side road food vendors, and other establishments [2]. They have been a popular option for consuming hot and cold beverages. They were initially developed in the early last century as a life-saving technology to control the spread of diseases through shared drinking cups. During the 1918 influenza and COVID-19 pandemics, disposable paper cups have been used more widely for hygiene purposes. Nowadays, they continue to be more popular due to their convenience and single-use design, which helps minimize the risk of cross-contamination [3]. However, their environmental impact has also become a global concern [1].

In 2023, the paper cup market in Gulf Cooperation Council (GCC) countries is valued at USD 350 million (which accounts for ~4% of the global market), and it is anticipated to grow to USD 440 million by 2029 [4]. The paper cups market, in the Kingdom of Saudi Arabia, grows significantly owing to the marked growth of the food and beverage service market as a result of increasingly relaxed social norms and high demand for prepared beverages for Hajj/Umrah, the airline industry, and catering for social celebrations and entertainment activities. In general, the Kingdom generates about 2.4 million tons of paper waste, constituting around 13% of the overall municipal solid waste [5]. Unfortunately, there is limited knowledge about the health and environmental impacts of paper cups consumed in the Kingdom. A literature survey conducted by the authors using Scopus and Web of Science databases with the keywords “paper cups” and “Saudi Arabia” has shown only one study that focused on investigating this topic [6].

Typically, the exterior layer of disposable paper cups, which represents 90–95% (by weight) of the cup, is made of paper, while the interior layer (5–10%) is mostly a hydrophobic polymeric thin layer [7]. Several organic and inorganic additives are commonly used to treat the cups in order to make them gain desired properties like flexibility, heat durability, color, and resistance to microbial activities [1,8,9,10,11]. It has been documented that potentially toxic elements and their compounds exist in plastic materials [8]. In our previous work, 38 chemical elements, including potentially toxic elements and natural radionuclides, were determined in some plastic products [9]. Chemical elements may be incorporated as functional additives, present as impurities in the raw materials used in polymeric substrate production, or remain as residues from the manufacturing processes [10,12,13]. Normally, raw materials are combined with inorganic nano-filler materials like metals, silicates, and carbonates to enhance the mechanical properties and increase the flexibility of the polymeric materials used in the food packing industry [11].

It has been confirmed that the use of disposable paper cups in hot beverage services can result in releases of a variety of harmful organic chemical substances and potentially toxic elements under high-temperatures (~85–90 °C), which may pose a significant risk to both the environment and human health [1,14,15]. Disposal of paper cups represents a serious environmental issue as they are made from materials that are resistant to natural degradation [16]. Moreover, the strong bond between the laminated polymeric films and paper fibers makes their separation not practically feasible, which hinders the recyclability of paper cups [17]. Additionally, converting paper cups into usable products such as catalysts [18], carboxymethylcellulose [19], and plastic–paper composites [20] is a temporary solution and does not provide full destruction of the material. Consequently, if not incinerated, paper cups are sent to landfills, where they remain for a long time, potentially causing a serious environmental concern. Thorough characterization of their chemical composition in terms of valuable potentially toxic and radioactive elements and organic and inorganic compounds is a crucial parameter for understanding and predicting the long-term environmental impacts of disposable paper cups. Additionally, assessing the potential for recovery of valuable elements from incineration residue is of significant economic importance.

At the University of Jeddah, we have been involved in investigating the fate and the environmental and health impacts of single-use paper cups. As a result, an extensive project has been launched to address this subject in order to support the decision-makers to adapt adequate measures to control the use of single-use products and monitor their health consequences and environmental toxicity. As an initial and important step, the present work aims to characterize the potentially toxic elements and naturally occurring radionuclides (^40^K, Th, and U) in the disposable single-use paper cups. This assessment is crucial for ensuring the safety and well-being of individuals who may be exposed to these materials, as well as for a deep understanding of their potential environmental and ecological risks. Paper cups of different decorative colors/paintings from different manufacturers were also examined for their elemental composition and natural radioactivity. Owing to its accuracy and multi-elemental analytical capabilities, the neutron activation analysis technique was chosen to perform the required elemental analysis of the investigated samples [8,9,21]. Moreover, the amount of CO_2_ emissions and the incineration residue were evaluated too.

## 2. Materials and Methods

### 2.1. Sampling

Samples of pristine paper cups were obtained from the local market, Jeddah, Saudi Arabia. In total, 14 paper cups from different manufacturers were sampled. Sampling of cups with different colors/decoration was considered. No special treatments of preparation processes were applied on the examined samples; only washing several times with de-ionized water was applied to remove any loose contaminants attached to the samples’ surfaces. To prevent any probable cross-contamination, ceramic scissors were employed to cut the paper cups under examination into small samples. Colored/decorated, different parts of the cup were also sampled. Each sample, weighing ~500 mg, was wrapped with high-purity polyethylene sheet and kept for further examinations.

### 2.2. Elemental Analysis Technique

The neutron activation analysis (NAA) method was employed for determining the elemental composition of the examined paper cups. The basic principle of the NAA is the bombardment of the investigated sample with neutrons to convert the stable isotopes into radioactive nuclides, followed by measuring the induced radioactivity employing a high-purity germanium (HPGe) detector to the resulting gamma-ray spectra. The obtained spectra are then interpreted in terms of qualitative and quantitative elemental composition. The detailed analysis protocol to determine the elemental composition using NAA was published in previous works [8,9,21]. In general, the samples were exposed to two neutron irradiation schemes. The first one consists of short-term irradiation (60 s) under the influence of thermal neutron flux of 3.7 × 10^12^ cm^−2^ s^−1^. Afterward, the irradiated materials were counted with the HPGe for 300 and 1500 s following decay intervals of 200 and 3600 s, respectively. This scheme is useful for determining elements that produce analytical radionuclides with relatively short half-lives upon neutron irradiation. On the other hand, employing the second neutron irradiation scheme enables the determination of elements through their relatively long-lived radionuclides. This process involves 6 h of irradiation at a thermal neutron flux of 2.3 × 10^12^ cm^−2^ s^−1^. A first measurement of 2 h was carried out for the irradiated samples after a decay interval of 4–6 days, followed by a second measurement of 4 h after a 21–28-day decay period. Table 1 shows a summary of the applied schemes for analyzing the examined samples.

An n-type HPGe detector (EG&G Ortec) was utilized for measuring the induced gamma radiations in the irradiated paper cups. The employed detector has a relative detection efficiency of 100% and an energy resolution of 2.15 keV at the ^60^Co line 1332.5 keV. It operates with the Gamma Vision software package (version 5.34), and the acquisition system incorporates the Maestro Multi-Channel Analyzer emulation software card, coupled with the HPGe through electronic modules. Detector calibration was performed using ^152^Eu and ^137^Cs calibration radioactive point sources supplied by Isotope Products Laboratories. The *k*_0_-IAEA software (version 10.11) was used to estimate the concentration levels of the detected elements in the examined cups, as detailed in previous studies [22].

The reliability of the analytical procedures was assessed by analyzing quality control reference materials (Oriental Basma Tobacco Leaves (INCT-OBTL-5), NIST SRM-1515 Apple Leaves) under the same conditions as the samples of examined paper cups. The analysis results of the reference materials were then assessed using two instruments, the relative *Bias*% and ζ−score [23].

(1) $$Bias\%=\frac{C_{m}-C_{r}}{C_{r}}\times 100$$

(2) $$\zeta -score=\frac{C_{m}-C_{r}}{\sqrt{u_{m}^{2}+u_{r}^{2}}}$$

where C and u indicate the concentration level and combined standard uncertainty, respectively, while the subscripts “*m*” and “*r*” represent the measured and certified values, respectively. Based on the policy of our lab, the accepted Bias% < ׀15%׀. On the other hand, ζ−score values can be interpreted according to the ISO/IEC 17043: 2010 B.4.1.1 as follows:

|ζ| ≤ 2 signifies that the result is acceptable.

2 < |ζ| < 3 serves as a cautionary indicator.

|ζ| ≥ 3 signifies unacceptable results (or indicates the need for an action).

### 2.3. CO_2_ Emission and Elements Enrichment in Incineration Ash

The examined paper cups were incinerated, and the ash percentage was calculated according to the following formula:

(3) $$Ash\%=\frac{mass\;of\;the\;ash}{dry\;mass\;of\;the\;cup}\times 100$$

The CO_2_ emission (kg/y of paper cups) from the incineration of paper cups, which has two main components (paper and plastic lining), can be calculated according to the following equation [24]:

(4) $${CO}_{2}-emission=\frac{44}{12}\times {(M}_{pc}\times \left[\left({WF}_{p}\times {CF}_{p}\times {OF}_{p}\right)+({WF}_{l}\times {CF}_{l}\times {OF}_{l})\right]$$

where *M_pc_*, *WF*, *CF*, *FCF*, and *OF* refer to the total annual amount of paper cup waste (kg/y), fraction of paper or plastic material, fractions of carbon, fraction of fossil carbon in dry paper cups, and oxidation factor, respectively. The subscripts “*p*” and “*l*” indicate the paper and lining plastic materials, respectively. The default values of these parameters are listed in Table S1 (Supplementary Data). The factor 44/12 refers to the conversion from C to CO_2_.

Element enrichment factors (*EF*) were estimated by normalizing their mass fractions in incineration ash (*C_ash_*) to the upper continental crust (*C_UCC_*) according to the following expression:

(5) $$EF=\frac{C_{ash}}{C_{UCC}}$$

### 2.4. Calculating Activity Concentration

The determined mass fractions *C_ash_* (mg/kg) of K, Th, and U as estimated from the ash fraction and NAA can be transformed into the corresponding activity concentrations *A_c_* (Bq/kg) using to the following equation [8]:

(6) $$A_{c}=C\frac{\theta N_{Av}\lambda }{M}$$

where *θ*, *N_Av_*, *λ*, and *M* refer to the natural isotopic abundance of the examined radionuclide, Avogadro’s number, the decay constant (s^−1^), and element atomic weight (g), respectively. Natural isotopic abundances of uranium isotopes ^235^U and ^238^U are 0.00720 and 0.99275, respectively, while they are 0.000117 and 1.0 for ^40^K and ^232^Th, respectively [25].

### 2.5. Radiological Health Risk Assessment

To assess the radiological risk associated with exposure to radionuclides in the incineration of paper cups, several radiological hazard indices were estimated. The examined indices include the absorbed dose rate *D_r_*, the annual effective dose equivalent *D_annual_*, and the excess lifetime cancer risk ELCR. These indices provide a comprehensive understanding of the potential health risks posed by the radionuclides present in the incineration of paper cups. By analyzing these indices, we can determine the level of radiological hazard and take appropriate measures to mitigate any potential risks.

The absorbed dose rate ($D_{r},\;nGy/h$) due to exposure to the examined radionuclides ^40^K, Th and U in the air at a distance of one meter from the examined ash materials can be defined according to the following equation [8,9]:

(7) $$D_{r}={0.0417\times A}_{c,K}+{0.604\times A}_{c,Th}+0.462\times A_{c,\;U}$$

where A_c,K_, A_c,Th_, and A_c,U_ refer to the specific radioactivity levels (Bq/kg) of ^40^K, Th, and U, respectively. These examined indices can be defined as the following:

The estimated *D_r_* can be translated into an annual effective dose equivalent (*D_annual_*) using a conversion factor of 0.7 Sv/Gy with outdoor and indoor occupancy factors of 0.2 and 0.8, respectively, as proposed by UNSCEAR [26]. The *D_annual_* in unit of Sv/y can be estimated from the following equation:

(8) $$D_{annual}=D_{r}\times T\times F$$

where T indicates the outdoor/indoor occupancy times, hour per year, and F is the absorbed dose to effective dose equivalent conversion factor (0.7 Sv/Gy). The following equations can be used to estimate the outdoor (D_out_) and the indoor (D_in_) annual effective doses using the above-mentioned occupancy factors:

(9) $$D_{out}(mSv/h)=D_{r}\;\times \;0.2\;\times \;1820\;\times \;0.7\;\times \;10^{-6}$$

(10) $$D_{in}(mSv/h)=D_{r}\;\times \;0.8\;\times \;1820\;\times \;0.7\times 10^{-6}$$

where 1820 h/y refers to the working time per year for an individual (7 h × 5 d × 52 w).

The excess lifetime cancer risk (ELCR) quantifies the likelihood of developing cancer over a lifetime due to radiation exposure. It can be estimated according to the following equation [27]:

(11) $$ELCR=D_{annual}\times DL\times RF$$

where DL is the average life duration (70 years), and RF is the fatal cancer risk per Sievert (risk factor, 0.05 Sv^−1^).

## 3. Results

### 3.1. Quality Control

**Figure 1:**
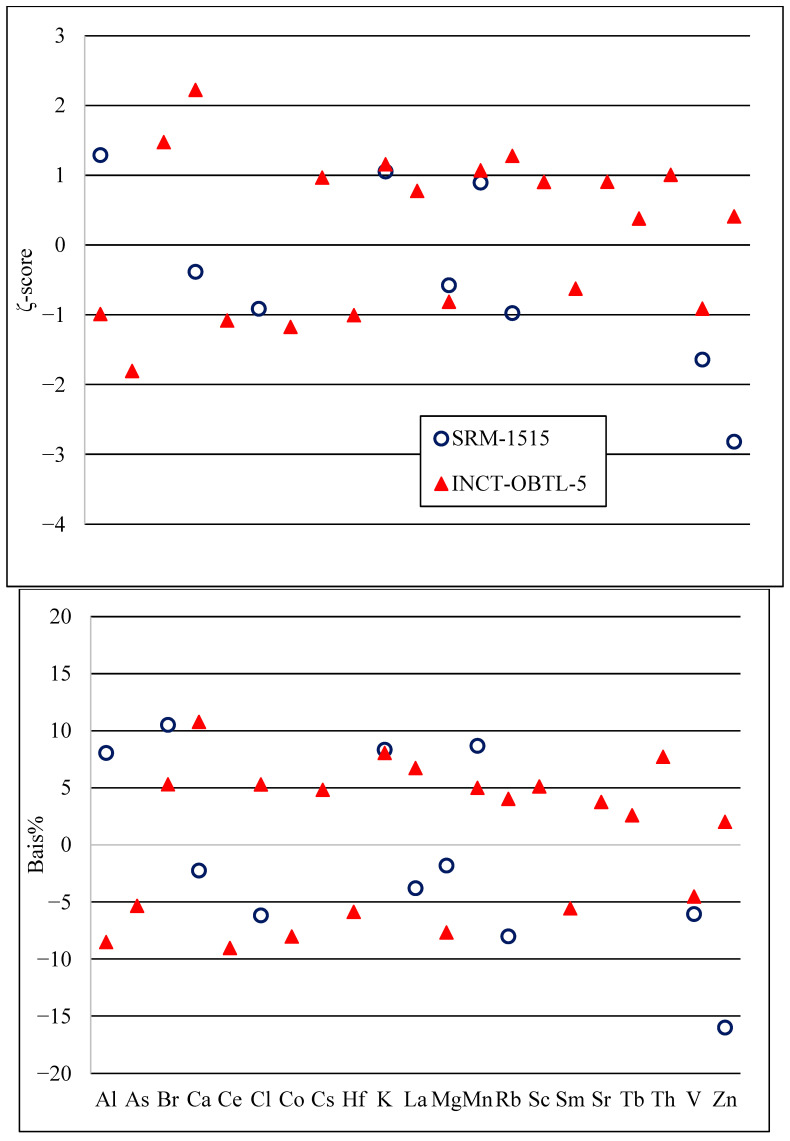
Evaluation of the analysis results of quality control reference materials.

To confirm the reliability of the applied NAA procedures, quality control materials (NIST SRM-1515 and INCT-OBTL-5) were treated in the same way as the samples of the investigated paper cups to confirm the validity and reliability. Figure 1 illustrates the assessment of the analysis results for these quality control materials using two instruments: the relative Bias% and the ζ−score. The ζ−score was not estimated for elements with mass fractions designated as “information values” without associated uncertainties.

Based on the observed values of the relative *Bias*% and the ζ−score, which fall within the acceptable limits, it can be noted that the determined elements show a good agreement between the measured and certified mass fractions. The only exception is Zn in SRM-1515, which has the highest deviation from the reference value by −16% and the highest ζ-score absolute value of 2.81. This discrepancy can be attributed to the spectral interference of the ^65^Zn gamma line at 1115 keV with ^46^Sc at 1120 keV. Nevertheless, as per the policy of our laboratory, the determined mass fractions are consistent with the reference values. This finding underscores the validity of the applied analytical procedures and affirms their suitability for analyzing the paper cups under investigation. Moreover, the consistent results obtained from different quality control materials further validate the robustness of the NAA protocol. Such consistency confirms that the employed analytical methods are appropriate for conducting reliable and accurate elemental analysis.

### 3.2. Elemental Composition of the Examined Paper Cups

The NAA technique enabled the accurate quantification of 35 elements, providing valuable insights into the elemental composition of the single-use paper cups under examination. Among the determined elements: three are naturally occurring radioactive elements: Th and U, as well as the radionuclide ^40^K (as it can be estimated from the determined potassium (K) content). Potentially toxic elements, including Al, Ba, Co, Cr, Cu, Mn, Mo, and V, were detected in all examined samples, exhibiting varied mass fractions. Moreover, two halogens (Br and Cl) as well as nine lanthanides were also identified. The median, associated uncertainties, minimum, and maximum mass fraction values of the determined elements are listed in Table 2. Generally, the mass fractions of the detected elements showed considerable variation overall, ranging from sub-nanogram per kilogram (ng/kg) to percentage levels. Notably, Mg, Al, and Ca were present in relatively high mass fractions, averaging (2.09 ± 0.595)%, (0.173 ± 0.389)%, and (0.171 ± 0.113)%, respectively. Furthermore, ten additional elements were determined at milligram per kilogram (mg/kg) levels, with mass fractions ranging from 2.73 mg/kg (for Zn) to 559 mg/kg (for Na). The abundance of these elements in the examined samples follows the descending order: Na > Fe > Ti > Cl > K > Mn > Sr > Cu > Ba > Zn. Conversely, the remaining 24 elements were detected at sub-milligram per kilogram (sub-mg/kg) levels, with the lowest mass fraction reported for Au, 0.524 ± 0.282 ng/kg.

**Figure 2:**
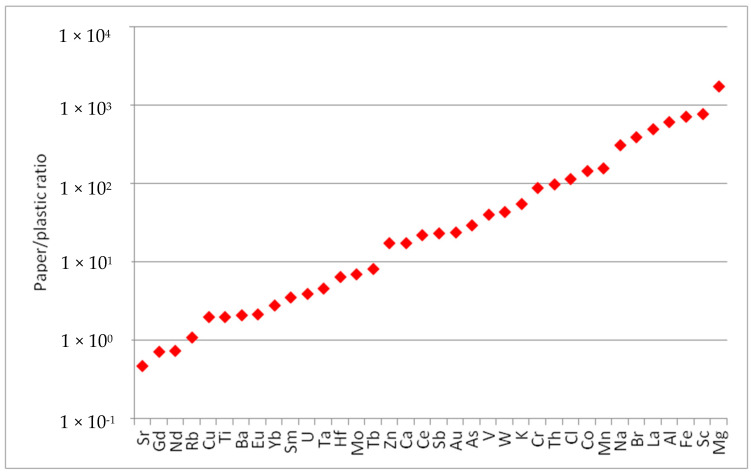
Comparison between the elemental compositions of paper and plastic cups.

In order to thoroughly grasp the concentration levels of the determined elements in examined paper cups, these were compared with the corresponding values determined in conventional plastic cups sourced from the local market. Figure 2 shows the ratios between the mass fractions of the determined elements in paper and plastic cups. The findings reveal that paper cups have significantly higher mass fractions of determined chemical elements except Sr, Gd, and Nd, which are lower in paper cups. This suggests that these elements are more prevalent in paper cups compared to plastic cups. The higher presence of these elements could be due to the materials and processes used in the manufacturing of paper cups. The differences in the elemental composition between paper and plastic cups can have various implications. For instance, the higher levels of potentially toxic elements (e.g., As, Ba, Cr, Cu, Fe, Mn, Mo, Sb, V, etc.) and radioactive elements (Th and U) in paper cups might affect their recyclability, environmental impact, and potential health risks. It has been reported that potentially toxic elements and other harmful chemical substances can be leached from paper cups and cause adverse effects on human health [1,6,15].

### 3.3. Element-Color Correlation

**Figure 3:**
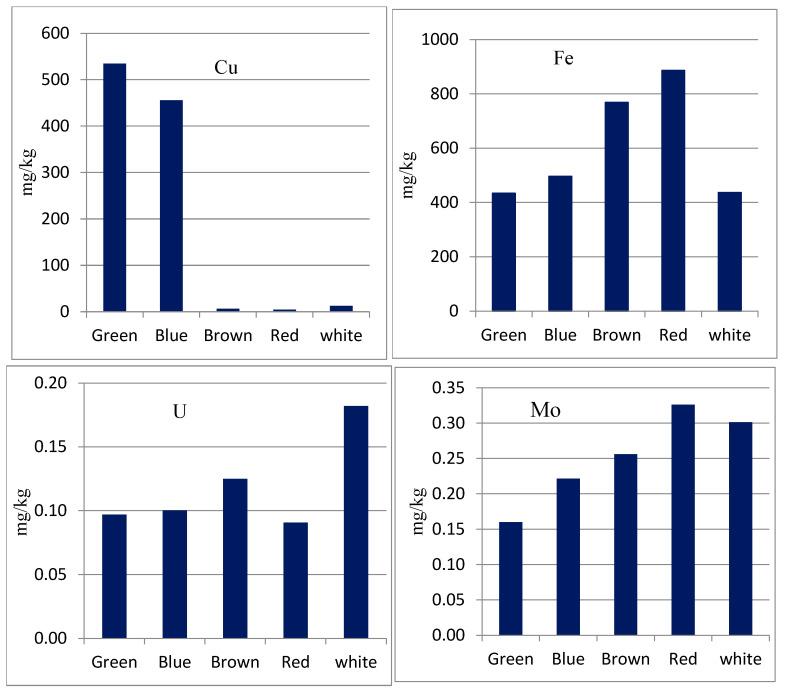
Elemental composition as a function of the color of the examined paper cup.

The color/painting of paper cups is often influenced by the dyes and pigments used in their production. These pigments and dyes may contain potentially toxic elements in their structure, which could also influence the overall safety and environmental impact of the paper cups. To assess the potentially toxic elements in the painted/decorated paper cups, the colored parts of each cup were cut and analyzed for their elemental composition. Pearson’s correlation approach was applied to measure the strength of the correlation between the color of test paper cups and their elemental composition. The obtained correlation coefficients are listed in Table S2 (Supplementary Data). It was found that some elements are correlated (to a certain extent) with the color of the cups. Figure 3 shows the concentration of selected elements as a function of the color of the investigated paper cups. It can be noted that blue and green colors are rich in Cu, while brown, yellow, and red colors are rich in Fe. For example, the Cu mass fractions in green and blue cups reach ~550 mg/kg compared to less than 10 mg/kg in plain paper cups. This can be attributed to the presence of these elements in the composition of the pigments commonly used in the paper cup industry, such as Phthalocyanine Green (C₃₂H₁₆N₈CuCl₁₆), Phthalocyanine Blue (C₃₂H₁₆N₈Cu), Red Iron Oxide (Fe₂O₃), etc. On the other hand, some other trace elements (ex: U) are rich in plain paper cups, which can be attributed to the raw materials and/or the manufacturing processes.

### 3.4. CO_2_ Emission from Incineration of Paper Cups and Element Enrichment

**Figure 4:**
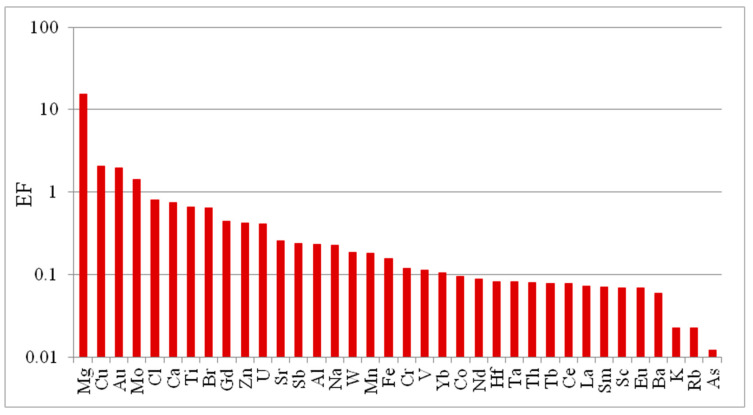
Enriched factors of elements in incineration ash of paper cups (based on the median values of determined mass fractions of these elements).

Due to the lack of an official estimate for the total annual waste of paper cups in the Kingdom, the annual amount of CO_2_ emissions from the incineration of paper cups cannot be estimated. Instead, the CO_2_ emission per kg of paper cup was estimated according to Equation (4). Taking into account the emission parameters listed in Table S1, the amount of CO_2_ emission per kg of paper cup waste was estimated at 1.77 kg/kg, which is slightly higher than the typical amount emitted from paper waste, 1.63 kg/kg. This can be attributed to the composition of paper cups, which include ~5–10% plastic materials [7]. Based on incineration experiments, it was found that the residual ash left after incineration of paper cups makes up 8–10% of the original mass of the paper cups. The elemental composition in the ash content was characterized in order to evaluate their environmental impact as well as to assess the potential of valuable elements recovery. Element enrichment factors were estimated by normalizing to the UCC [28]. As observed from the data presented in Figure 4, elements exhibit varying enrichment factors ranging from 0.01 up to 15.5 (based on the median value of the determined mass fractions). In relation to the UCC, only the elements Mg, Cu, Au, and Mo showed enrichment factors greater than unity (with EF values of 15.5, 2.1, 2.0, and 1.4, respectively), while the other thirty-two elements can be considered as depleted elements in the incineration ash.

The relatively high concentrations of these four elements in the incineration ash of paper cups present both challenges and opportunities. Environmentally, careful management is required to control soil and water contamination. Economically, these elements are valuable, and their recovery can be profitable and support sustainable practices by reducing the need for new mining activities and promoting recycling of materials. Magnesium is beneficial for soil quality and essential for plant growth, and it is used in various industrial applications, including the production of lightweight alloys, which are essential for automotive and aerospace industries. Copper, molybdenum, and gold have medium to high-market values, and they are used in different industrial applications. To assess the potential of future recovery of these elements, their concentrations in the incineration ash are evaluated against the standards of ores cut-off grade and minimum industrial grade [29,30,31].

Table 3 shows a direct comparison between the ore cut-off grades of these elements and their mass fractions in the incineration ash of paper cups. In general, Mg is the only element that can be economically recovered, as its concentration (median: 23.5 ± 6.6%) is well above the ore cut-off grade of 12%. On the other hand, Cu, Au, and Mo occur in average concentration levels significantly below their ore cut-off grade. Shedding more light on the distribution of Cu in the different colored cups (Figure 3), it can be observed that it shows relatively high mass fractions in the incineration ashes of green and blue cups at 0.59% and 0.51%, respectively, which are slightly higher than the ore cut-off grade of 0.5%. These findings reveal that the incineration ash of paper cups can be considered a promising source for recovering the valuable elements Mg and Cu (from green and blue cups).

### 3.5. Activity Concentration and Radiological Impact of Incineration Ash

**Figure 5:**
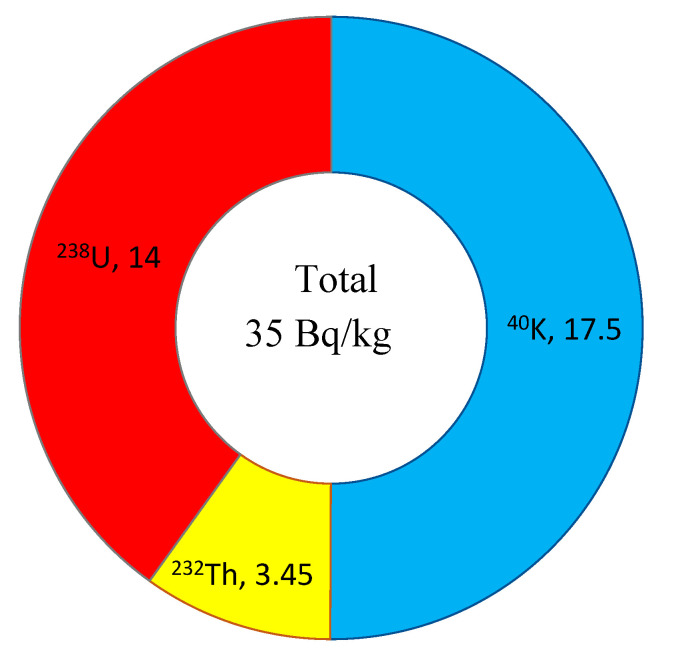
Activity concentration in the incineration ash of paper cups.

The naturally occurring radionuclides ^40^K, Th, and U were detected in all examined paper cups, and their activity concentrations in the incineration ash were estimated from their determined mass fractions according to Equation (6). The specific activities of these radionuclides are presented in Figure 5. The median of the total detected radioactivity is 35 Bq/kg. It can be noted that ^40^K accounts for around one half of the total detected activity in the examined samples, while Th and U account for 10% and 40%, respectively. Although this radioactivity is relatively low, it is still important to monitor and manage it, especially given the large volume of paper cups disposed of in Saudi Arabia and globally.

The various radiological hazard indices mentioned above can be used to assess the radiological risk from exposure to paper cups. The values of D_r_, D_annual_, and ELCR as estimated according to Equations (7), (8), and (11), respectively, are presented in Table 4. The dose rate (D_r_) of 24.3 nGy/h is relatively low and well below the population-weighted world average of 55 nGy/h, indicating a minimal level of radiation exposure. The annual dose (D_annual_) of 31 µSv/y is also quite low and significantly lower than the recommended upper dose limits for the public (1 mSv/y) and workers (20 mSv/y). The excess lifetime cancer risk (ELCR) of 2.17 × 10^−5^ is very small, suggesting that the risk of developing cancer due to this level of radiation exposure is negligible. This estimated ELCR value is much lower than the typical safety threshold for radiological risk, which is often considered to be 1 × 10^−4^.

## 4. Conclusions

This study successfully applied neutron activation analysis to evaluate the elemental composition and radiological impact of single-use paper cups. The quality control measures, including the use of standard reference materials, confirmed the validity and reliability of the analytical procedures. The analysis identified 36 elements in the paper cups, including naturally occurring radionuclides ^40^K, Th, and U, as well as potentially toxic elements such as Al, Ba, Co, Cr, Cu, Mn, Mo, and V.

Incineration of paper cups generates a slightly higher amount of CO_2_ than the conventional paper; the incineration ash represents about 8–10% of the original mass. In general, the incineration residue shows significantly high concentration levels of Cu (~0.5–0.6% in green and blue cups) and Mg (23.5%), which are above their ore cut-off grades, making it a promising source for recovery of these valuable elements. Additionally, attention should be given to controlling the use of potentially toxic elements (ex.: Cu) in the coloring of paper cups.

The median total radioactivity was found to be 35 Bq/kg. Although the radioactivity levels are relatively low, they should be considered in the context of the large volume of paper cup waste generated globally. The radiological risk assessment, based on various hazard indices, indicated minimal risk to human health, with the dose rate (D_r_) of 24.3 nGy/h and the annual dose (D_annual_) of 31 µSv/y being well below the recommended limits. The excess lifetime cancer risk (ELCR) of 2.17 × 10^−5^ is significantly lower than the typical safety threshold.

Overall, the findings underscore the importance of monitoring and managing the radiological and toxicological impact of paper cup waste, especially given the widespread use and disposal of these products in landfills where there may be leaching of various elements. Further research is recommended to explore the long-term environmental and health implications of the detected elements and radionuclides in paper cups.

## Figures and Tables

**Table 1:** Summary of the applied analysis protocol.

Scheme	Irradiation Period	Decay Interval	Measuring Time	Determined Elements
Short irradiation	2 min	3 min	5 min	Al, Ca, Mg, Ti, V, and Cu
		60 min	30 min	Na, Cl, K, and Mn,
Long irradiation	6 h	2–5 d	2 h	As, Au, Br, La, Mo, Sb, Sm, U, and W
		21–28 d	4 h	Ba, Ce, Cr, Co, Cs, Eu, Fe, Gd, Hf, Nd, Sc, Sr, Ta, Tb, Th, Yb, and Zn

**Table 2:** Elemental composition of the examined paper cups.

Element	Mass Fraction, mg/kg			
	Median	STDEV	Minimum	Maximum
Mg	2.09 × 10^4^	5.95 × 10^3^	1.01 × 10^4^	2.99 × 10^4^
Al	1.73 × 10^3^	3.89 × 10^3^	9.75 × 10^2^	1.00 × 10^4^
Ca	1.71 × 10^3^	1.13 × 10^3^	1.14 × 10^3^	4.74 × 10^3^
Na	5.59 × 10^2^	1.84 × 10^2^	2.71 × 10^2^	9.49 × 10^2^
Fe	4.94 × 10^2^	5.96 × 10^2^	1.52 × 10^2^	1.85 × 10^3^
Ti	2.26 × 10^2^	1.75 × 10^2^	9.10 × 10^0^	6.63 × 10^2^
Cl	2.07 × 10^2^	7.98 × 10^1^	1.19 × 10^2^	4.10 × 10^2^
K	4.75 × 10^1^	9.73 × 10^1^	5.10 × 10^0^	2.74 × 10^2^
Mn	1.27 × 10^1^	5.31 × 10^0^	7.13 × 10^0^	2.24 × 10^1^
Sr	7.34 × 10^0^	1.38 × 10^1^	2.45 × 10^0^	4.18 × 10^1^
Cu	5.18 × 10^0^	2.31 × 10^2^	2.15 × 10^0^	5.71 × 10^2^
Ba	3.37 × 10^0^	6.06 × 10^0^	1.14 × 10^0^	2.19 × 10^1^
Zn	2.73 × 10^0^	2.53 × 10^0^	5.84 × 10^−1^	9.63 × 10^0^
V	9.96 × 10^−1^	3.33 × 10^0^	5.50 × 10^−2^	7.87 × 10^0^
Cr	9.83 × 10^−1^	7.17 × 10^0^	4.77 × 10^−1^	1.63 × 10^1^
Br	5.24 × 10^−1^	1.73 × 10^0^	2.84 × 10^−1^	6.98 × 10^0^
Ce	4.42 × 10^−1^	1.74 × 10^0^	1.16 × 10^−1^	4.49 × 10^0^
Nd	2.14 × 10^−1^	5.98 × 10^−1^	3.25 × 10^−2^	1.66 × 10^0^
La	2.01 × 10^−1^	8.95 × 10^−1^	4.61 × 10^−2^	2.33 × 10^0^
Mo	1.73 × 10^−1^	1.34 × 10^−1^	3.75 × 10^−2^	4.88 × 10^−1^
Rb	1.71 × 10^−1^	2.95 × 10^−1^	3.75 × 10^−2^	8.28 × 10^−1^
Gd	1.60 × 10^−1^	1.26 × 10^0^	3.15 × 10^−2^	4.85 × 10^0^
Co	1.48 × 10^−1^	3.08 × 10^−1^	5.09 × 10^−2^	7.54 × 10^−1^
U	1.00 × 10^−1^	6.04 × 10^−2^	7.85 × 10^−2^	2.57 × 10^−1^
Sc	8.63 × 10^−2^	4.67 × 10^−1^	4.80 × 10^−3^	1.15 × 10^0^
Th	7.62 × 10^−2^	5.10 × 10^−1^	4.61 × 10^−3^	1.17 × 10^0^
Hf	3.91 × 10^−2^	6.88 × 10^−2^	6.57 × 10^−3^	1.93 × 10^−1^
W	3.15 × 10^−2^	7.24 × 10^−2^	7.86 × 10^−3^	2.12 × 10^−1^
Sm	3.00 × 10^−2^	8.18 × 10^−2^	9.27 × 10^−3^	2.12 × 10^−1^
As	2.68 × 10^−2^	1.10 × 10^−2^	8.50 × 10^−3^	4.14 × 10^−2^
Yb	1.91 × 10^−2^	1.37 × 10^−2^	6.34 × 10^−3^	5.50 × 10^−2^
Sb	9.06 × 10^−3^	9.26 × 10^−3^	3.53 × 10^−3^	3.05 × 10^−2^
Ta	6.58 × 10^−3^	2.96 × 10^−2^	1.40 × 10^−3^	7.45 × 10^−2^
Eu	6.15 × 10^−3^	9.96 × 10^−3^	3.17 × 10^−3^	3.37 × 10^−2^
Tb	4.97 × 10^−3^	6.23 × 10^−3^	1.13 × 10^−3^	2.27 × 10^−2^
Au	2.66 × 10^−4^	2.88 × 10^−4^	1.14 × 10^−4^	1.19 × 10^−3^

**Table 3:** Comparing the median mass fraction of examined valuable elements in incineration ash with ore cut-off and minimum industrial grades.

Elements	Mass Fraction, %	
	Incineration Ash	Cut-Off Grade
Mg	23.2	12
Cu	0.006	0.5
	In green cups: 0.59	
	In blue cups: 0.51	
Au	2.96 × 10^−7^	5 × 10^−5^
Mo	1.9 × 10^−4^	0.10

**Table 4:** Radiological hazard parameters.

*D_r_*, nGy	*D*_annual_, µSv/y	ELCR
24.3	31 (*D_in_* = 6.2; *D_out_* = 24.8)	2.17 × 10^−5^

## Data Availability

The data presented in this study are available on request from the corresponding author.

## Supplementary Materials

The following supporting information can be downloaded at: https://www.mdpi.com/article/10.3390/toxics13030179/s1. Table S1. Default total carbon content, fossil carbon fraction, and oxidation state of paper and plastic materials. Table S2. Pearson correlation coefficient.

## Abbreviations

The following abbreviations are used in this manuscript:

NAA	Neutron Activation Analysis
ELCR	Excess Lifetime Cancer Risk
NIST	National Institute of Standards and Technology
