# Supplementary material for: Disposable Paper Cups: A Study on Potentially Toxic Elements, Radiological Impact, and Feasibility of Valuable Elements Recovery
Source: Toxics. 2025 Feb 28;13(3):179. doi: 10.3390/toxics13030179 (PMC11946655; doi:10.3390/toxics13030179)
Supplement: Supplementary file 1 [file toxics-13-00179-s001.zip › toxics-3481921-supplementary.pdf]

Supplementary data

Table S1. Default total carbon content, fossil carbon fraction, and oxidation state of paper and plastic materials

Material	Total carbon, %	Fossil carbon, %	Oxidation factor, %
Paper	46	1	100
Plastic	75	100	100

Table S2. Pearson correlation coefficient

Element	Correlation coefficient	Element	Correlation coefficient
Mg	0.512	Gd	-0.1732
Al	0.040	Mo	-0.929
Ca	-0.318	La	0.193
Na	0.043	Rb	-0.540
Fe	0.602	Co	0.038
Ti	-0.353	U	-0.67665
Cl	0.408	Sc	0.149
K	-0.049	Th	0.269
Mn	0.420	W	0.249
Sr	0.039	Hf	0.289
Cu	0.894	Sm	0.345
Ba	0.313	As	0.327
Zn	0.654	Yb	-0.606
V	0.0639	Sb	-0.164
Cr	0.615	Ta	0.404
Br	-0.199	Eu	0.024
Ce	0.138	Tb	-0.326
Nd	-0.018	Au	0.356
